# Identification and prognostic value of metabolism-related genes in gastric cancer

**DOI:** 10.18632/aging.103838

**Published:** 2020-09-11

**Authors:** Fang Wen, Jiani Huang, Xiaona Lu, Wenjie Huang, Yulan Wang, Yingfeng Bai, Shuai Ruan, Suping Gu, Xiaoxue Chen, Peng Shu

**Affiliations:** 1 Nanjing University of Chinese Medicine, Nanjing 210023, Jiangsu Province, China; 2 Department of Oncology, Affiliated Hospital of Nanjing University of Chinese Medicine, Nanjing 210029, Jiangsu Province, China; 3 Department of Oncology, Jiangsu Province Hospital of Chinese Medicine, Nanjing 210029, Jiangsu Province, China; 4 Department of Hematology, Jiangsu Province Hospital of Chinese Medicine, Affiliated Hospital of Nanjing University of Chinese Medicine, Nanjing 210029, Jiangsu Province, China; 5 College of Traditional Chinese Medicine, College of Integrated Traditional Chinese and Western Medicine, Nanjing University of Chinese Medicine, Nanjing 210023, China

**Keywords:** gastric cancer, TCGA, GEO, metabolism, prognostic model

## Abstract

Gastric cancer (GC) is one of the most commonly occurring cancers, and metabolism-related genes (MRGs) are associated with its development. Transcriptome data and the relevant clinical data were downloaded from The Cancer Genome Atlas and Gene Expression Omnibus databases, and we identified 194 MRGs differentially expressed between GC and adjacent nontumor tissues. Through univariate Cox and lasso regression analyses we identified 13 potential prognostic differentially expressed MRGs (PDEMRGs). These PDEMRGs (CKMT2, ME1, GSTA2, ASAH1, GGT5, RDH12, NNMT, POLR1A, ACYP1, GLA, OPLAH, DCK, and POLD3) were used to build a Cox regression risk model to predict the prognosis of GC patients. Further univariate and multivariate Cox regression analyses showed that this model could serve as an independent prognostic parameter. Gene Set Enrichment Analysis showed significant enrichment pathways that could potentially contribute to pathogenesis. This model also revealed the probability of genetic alterations of PDEMRGs. We have thus identified a valuable metabolic model for predicting the prognosis of GC patients. The PDEMRGs in this model reflect the dysregulated metabolic microenvironment of GC and provide useful noninvasive biomarkers.

## INTRODUCTION

Gastric cancer (GC) is one of the most common gastrointestinal cancers, with a high incidence in East Asian countries. The pathogenesis of GC is a multifactorial and multi-step process [1]. GC is diagnosed using endoscopy, biopsy, and pathology. Its five-year survival rate is related to the stage of the disease at diagnosis [2]. Early diagnosis has increased due to the application of advanced detection methods, but the prognosis of patients with advanced GC remains very poor [3, 4]. The occurrence of GC reflects the abnormal regulation of tumor-related genes [1, 5, 6]. Understanding the molecular mechanisms underlying GC will facilitate the diagnosis and enhance the treatment of GC, and the development of biomarkers for early detection will improve the prognosis of GC patients.

Dysregulation of the metabolic environment in the body plays a key role in cancer. The Warburg effect is a form of glycolysis that occurs in tumors in an aerobic environment [7]. One study found that a long non-coding RNA, lncRNA-MACC1, can enhance the Warburg effect in GC cells and up-regulate expression of glycolytic enzymes [8]. There are also changes in amino acid metabolism in patients with GC; the levels of cysteine, serine, isoleucine, tyrosine, and valine are increased [9, 10]. Exploring these metabolic changes in cancer may yield new treatments. In recent years, various metabolic enzymes and their products have become important as potential drug targets [11–13]. Drugs developed for the treatment of metabolic disorders may also be effective in the treatment of some cancers [14]. To study the prognostic utility of metabolism-related genes (MRGs) in GC, we established a GC prognostic risk model and studied its clinical application.

## RESULTS

### Identification of PDEMRGs in GC

**Figure 1:**
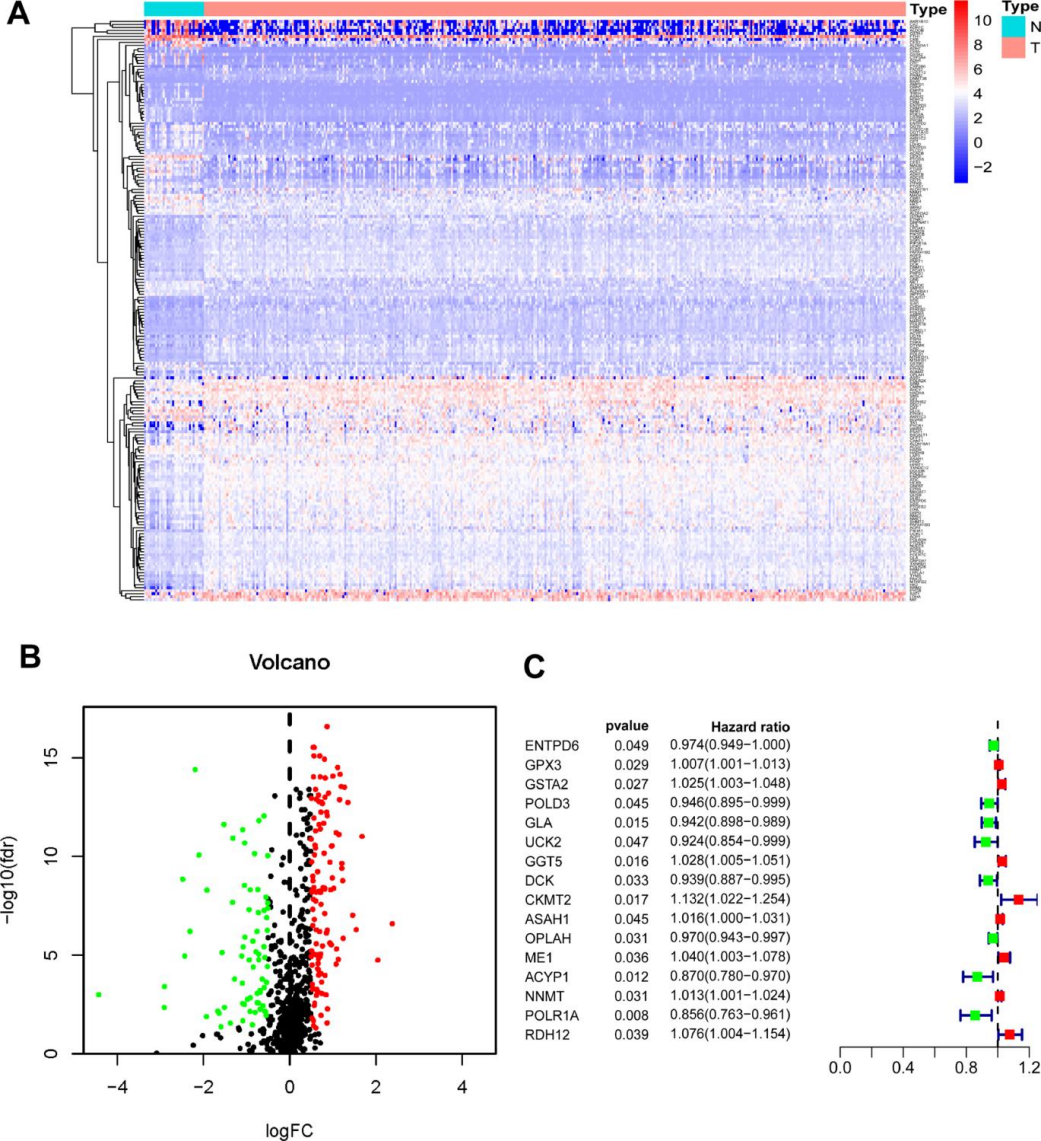
**Identification of PDEMRGs.** (**A**) Heatmap of DEMRGs: the red to blue spectrum signifies high to low gene expression. (**B**) Volcano plot of DEMRGs: red indicates upregulated DEMRGs, green indicates downregulated DEMRG, and black indicates DEMRGs that were not significantly differentially expressed. (**C**) Forrest plot of PDEMRGs: The red represents high-risk genes (hazard ratios, HR > 1); the green represents low-risk genes (HR < 1).

Four hundred and seven mRNA samples (375 GC tissues and 32 adjacent nontumor tissues) were analyzed in The Cancer Genome Atlas (TCGA). Through the Wilcoxon signed-rank test, 194 differentially expressed MRGs (DEMRGs) were obtained, including 122 up-regulated genes and 72 down-regulated genes of GC tissues compared with adjacent nontumor tissues (Figure 1A, 1B).

To determine the prognostic DEMRGs (PDEMRGs), univariate Cox regression analysis was used to screen the expression of DEMRGs in a training cohort. Sixteen DEMRGs (8 high-risk genes and 8 low-risk genes) were identified to be related to the overall survival (OS) of GC patients (Figure 1C).

### Establishment and validation of the prognostic risk model

**Figure 2:**
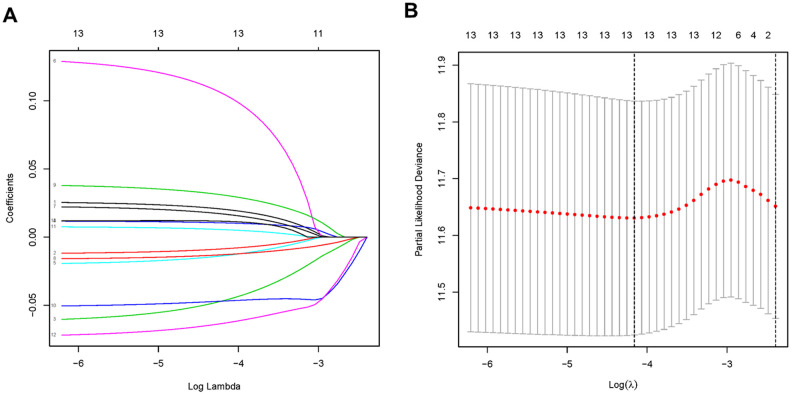
**PDEMRGs selected by lasso regression.** (**A**) Constructing the lasso coefficient prediction model. (**B**) Selecting variables in lasso regression with minimum criteria by 1000 times cross-validation.

Lasso regression was performed to remove PDEMRGs that are related to each other to prevent the model from overfitting (Figure 2A, 2B). We obtained 13 candidate PDEMRGs (risk genes) to construct the prognostic risk model (Table 1). CKMT2, ME1, GSTA2, ASAH1, GGT5, RDH12, and NNMT were identified as high-risk genes, while POLR1A, ACYP1, GLA, OPLAH, DCK, and POLD3 were identified as low-risk genes.

**Table 1:** Lasso Cox analysis.

**Gene**	**Full name of gene**	**Coefficient**	**Metabolism-related KEGG pathways**
CKMT2	Creatine kinase, mitochondrial 2	0.07837	Amino acid metabolism; Arginine and proline metabolism
ME1	Malic enzyme 1	0.02443	Lipid metabolism; Pyruvate metabolism;
GSTA2	Glutathione S-transferase alpha 2	0.01516	Other amino acids metabolism; Glutathione metabolism Xenobiotics biodegradation and metabolism; Metabolism of xenobiotics by cytochrome P450; Drug metabolism - cytochrome P450; Drug metabolism - other enzymes
ASAH1	N-acylsphingosine amidohydrolase 1	0.01175	Lipid metabolism; Sphingolipid metabolism
GGT5	Gamma-glutamyl transferase 5	0.00916	Lipid metabolism; Arachidonic acid metabolism; other amino acids metabolism; Taurine and hypotaurine metabolism; Glutathione metabolism
RDH12	Retinol dehydrogenase 12	0.00904	Cofactors and vitamins metabolism; Retinol metabolism
NNMT	Nicotinamide N-methyltransferase	0.00346	Cofactors and vitamins metabolism; Nicotinate and nicotinamide metabolism
POLR1A	RNA polymerase I subunit A	-0.05663	-
ACYP1	Acylphosphatase 1	-0.04524	Carbohydrate metabolism; Pyruvate metabolism
GLA	Galactosidase alpha	-0.03499	Galactose metabolism; Glycosphingolipid metabolism; Sphingolipid metabolism
OPLAH	5-oxoprolinase (ATP-hydrolysing)	-0.0105	Other amino acids metabolism; Glutathione metabolism
DCK	Deoxycytidine kinase	-0.00875	Nucleotide metabolism; Pyrimidine metabolism; Purine metabolism
POLD3	DNA polymerase delta 3	-0.00575	Pyrimidine metabolism

To study the role of the risk model in predicting the overall survival (OS) of GC patients, we used the expression levels of genes and regression coefficients to calculate the risk score for each patient. The risk score = (0.0152×expression of GSTA2) – (0.0058×expression of POLD3) – (0.0350×expression of GLA) + (0.0092×expression of GGT5) – (0.0088× expression of DCK) + (0.0784×expression of CKMT2) + (0.0117×expression of ASAH1) – (0.0105×expression of OPLAH) + (0.0244×expression of ME1) – (0.0452×expression of ACYP1) + (0.0035×expression of NNMT) – (0.0566×expression of POLR1A) + (0.0090×expression of RDH12).

**Figure 3:**
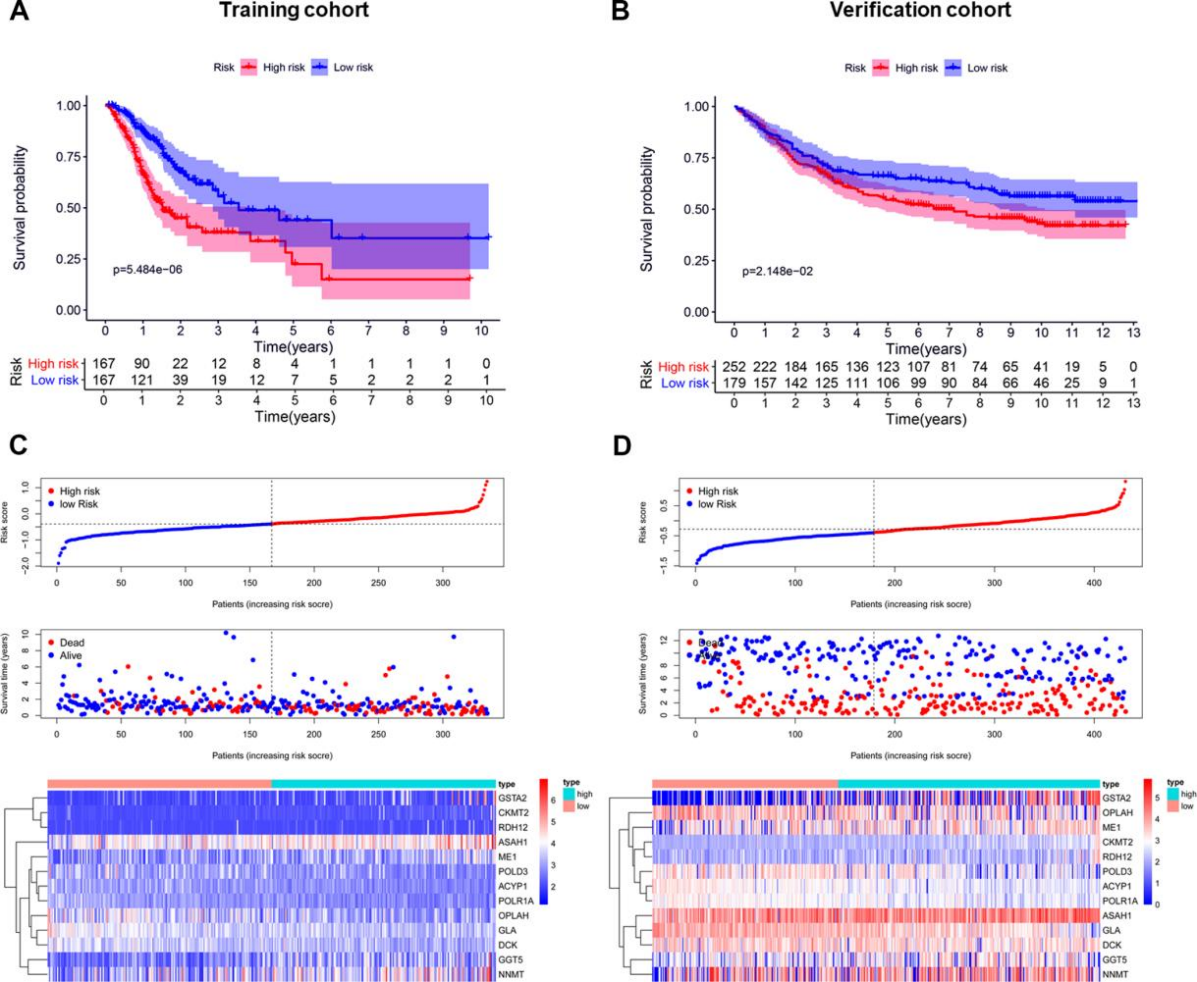
**Establishment and validation of the prognostic risk model.** (**A**, **B**) Kaplan-Meier curve analysis of the high-risk and low-risk groups. (**C**, **D**) From top to bottom=Risk score distribution of patients. Survival status scatter plots of patients. Expression patterns of risk genes.

Patients in the training cohort were divided into a high-risk (n=167) and a low-risk group (n=167) by the median risk score. To identify the prognostic difference between them we made a Kaplan-Meier curve. The OS was poorer in the high-risk group than the low-risk group (*p* < 0.001) (Figure 3A). We ranked the risk score of patients in the TCGA dataset (training cohort). The dot chart showed the survival state of patients and a heat map described the expression pattern of the high-risk and low-risk genes in the two groups (Figure 3C). Seven high-risk genes (CKMT2, ME1, GSTA2, ASAH1, GGT5, RDH12, and NNMT) were up-regulated, while six low-risk genes (POLR1A, ACYP1, GLA, OPLAH, DCK, and POLD3) were down-regulated. The risk genes showed opposite expression patterns for patients with low-risk scores. The prognostic model was validated in the Gene Expression Omnibus dataset (GEO, verification cohort), and the results were consistent with the training group (Figure 3B, 3D).

These results indicated that this risk model can accurately predict the prognosis for GC patients.

### Independent prognostic value of the risk model

We performed univariate and multivariate Cox regression analysis to determine if the risk score generated by the prognostic model was independent of other clinical indices.

**Figure 4:**
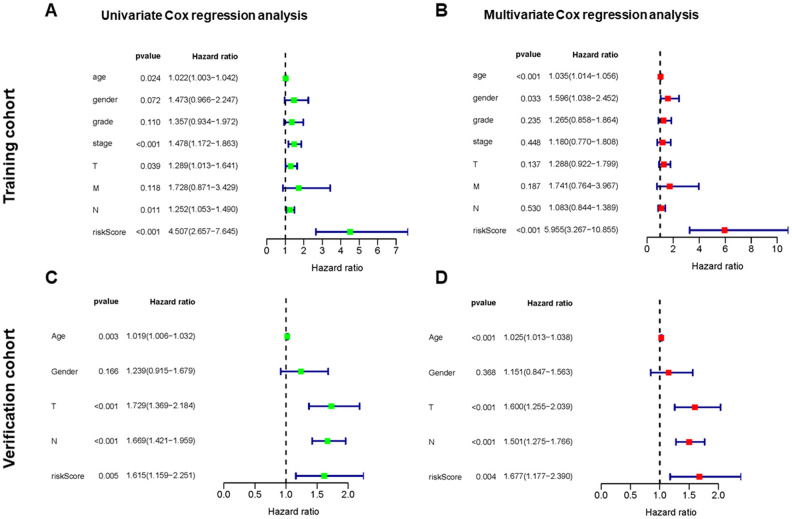
**Independent value of the prognostic risk model.** (**A**, **B**) Forrest plots of the univariate and multivariate Cox regression analysis in training cohort. (**C**, **D**) Forrest plot of the univariate and multivariate Cox regression analysis in verification cohort.

In the training cohort, univariate Cox regression analysis representing age, stage, tumor (T), node (N), and risk score were significantly correlated with the OS (*p* < 0.05) (Figure 4A). Multivariate Cox regression analysis indicated the variables of age, gender, and risk score were independently correlated with the OS (*p* < 0.05) (Figure 4B). In the verification cohort, univariate and multivariate Cox regression analysis showed the variables of age, T, N, and risk score were significantly associated with the OS (*p* < 0.05) (Figure 4C, 4D). The results indicated that the risk model could serve as an independent prognostic factor independent of other clinical indices.

**Figure 5:**
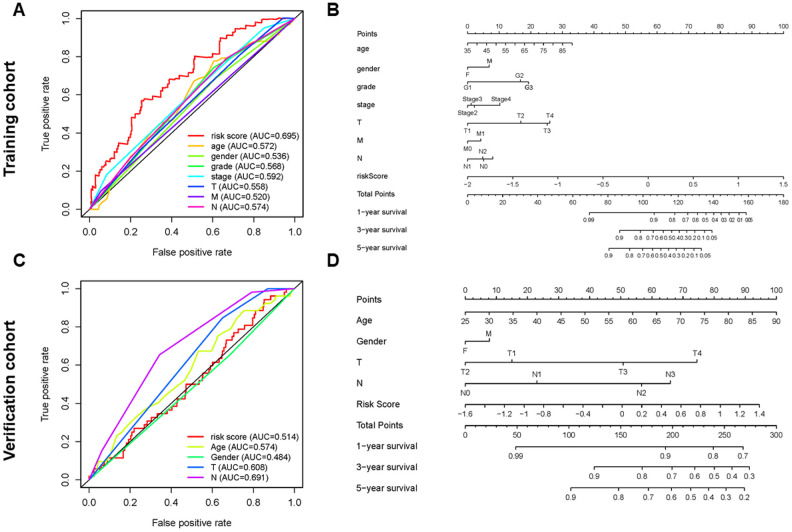
**Establishment of ROC curves and nomograms.** (**A**, **C**) ROC curves (receiver operating characteristics) of the risk score and other clinical indices. (**B**, **D**) The nomogram was established based on the independent prognosis model.

The risk score was more precise than other clinical indices. The AUCs (area under the curve) of the training cohort at risk score, age, gender, T, and N were 0.695, 0.572, 0.536, 0.558 and 0.574, respectively (Figure 5A). However, the risk score in the verification cohort is not the largest, which is not consistent with the result of the training cohort. This may be related to the relatively small sample size of the verification cohort (Figure 5C). To better predict the prognosis of GC patients, we established a nomogram model that accurately predicted the OS at 1, 3, and 5 years based on the variables associated with OS (age, gender, grade, stage, T, N, metastasis (M) and risk score) (Figure 5B, 5D).

### Gene set enrichment analyses

**Figure 6:**
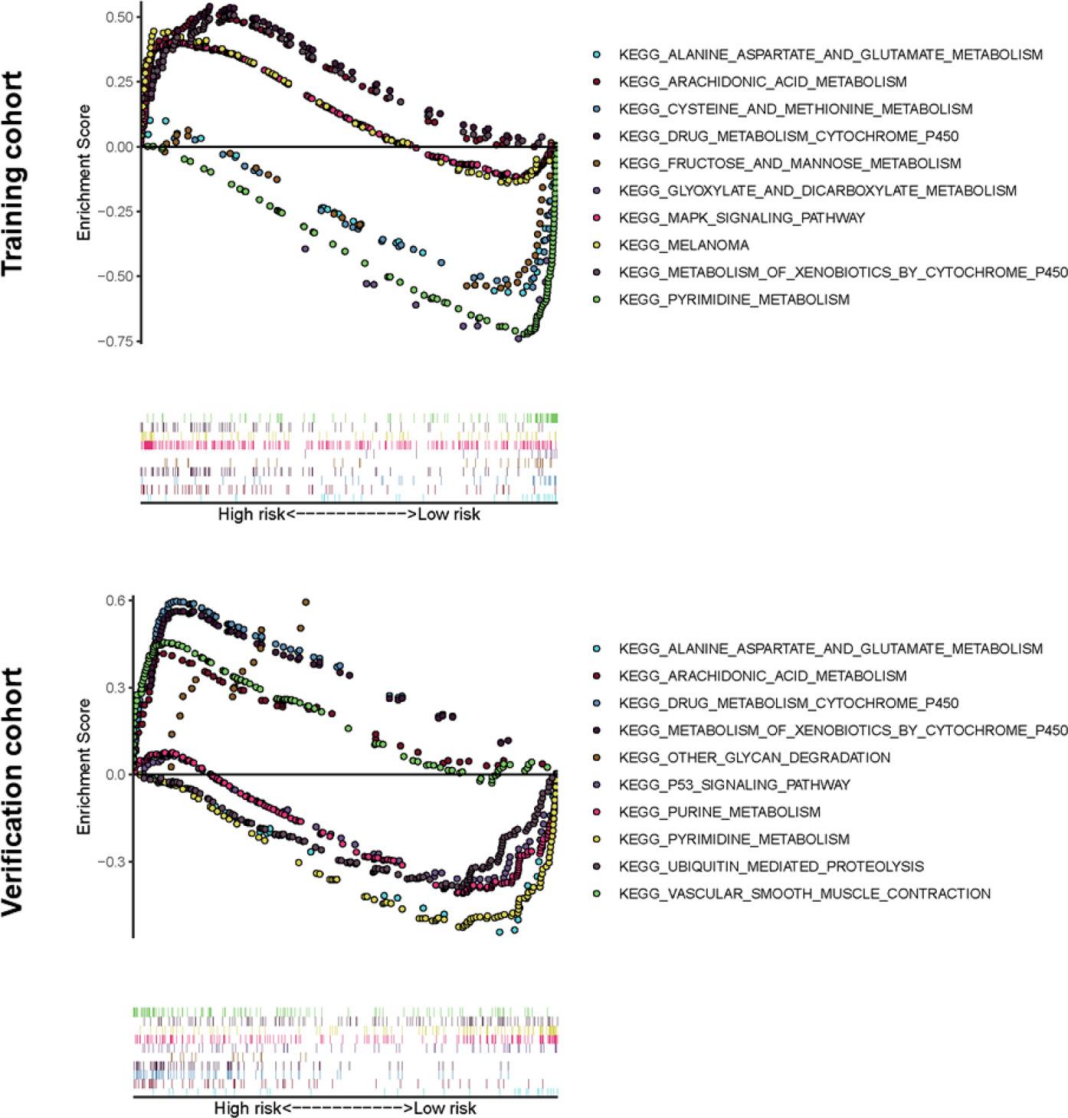
**Significantly enriched KEGG pathways in training or verification cohort by GSEA.** Above the horizontal axis indicated the pathways are in the high-risk group, and below the horizontal axis indicated that the pathways are in the low-risk group.

Gene Set Enrichment Analysis software (GSEA) was used and identified 60 significantly enriched Kyoto Encyclopedia of Genes and Genomes (KEGG) pathways in the training cohort or verification cohort (Nominal *p*-value < 0.05). The majority of enrichment pathways were associated with metabolism, including arachidonic acid metabolism, drug metabolism by cytochrome p450, xenobiotics metabolism by cytochrome p450, pyrimidine and purine metabolism, glyoxylate and dicarboxylate metabolism, alanine aspartate and glutamate metabolism, cysteine and methionine metabolism, and fructose and mannose metabolism. Physiological processes such as glycan degradation and ubiquitin-mediated proteolysis significantly inhibit the occurrence and development of cancer, and there are common signaling pathways in cancer, MAPK signaling pathway, and the p53 signaling pathway (Figure 6).

### Clinical utility of the PDEMRGs

**Figure 7:**
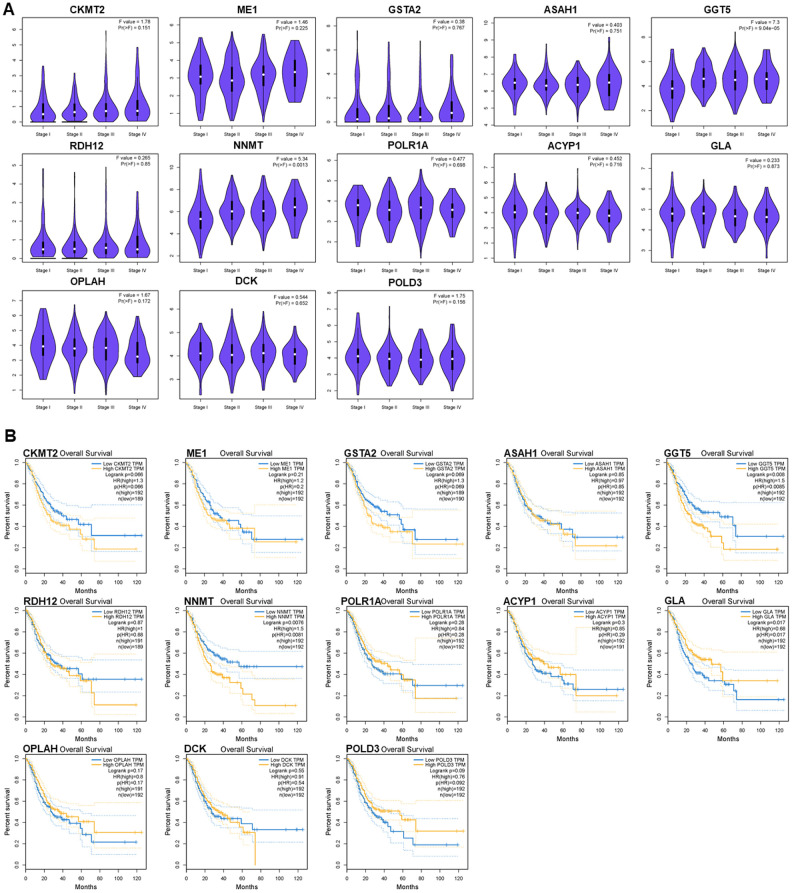
**Relationships of the PDEMRGs with pathological stage and survival time.** (**A**) The violin pot shows the expression levels of GGT5 and NNMT is significantly correlated with the pathological stage. (**B**) Survival plot indicates that the expression levels of GGT5, NNMT, and GLA are significantly correlated with OS.

Exploration of the PDEMRGs during GC clinical progression indicated that the levels of GGT5 and NNMT were increased with clinical stage. This correlation between the expression levels of these two genes and GC progression may be useful in GC diagnosis (Figure 7A).

Survival analysis indicated that the expression of GGT5, NNMT, and GLA had a significant association with patient survival (*P* < 0.05). Higher expression (yellow line) of GGT5 and NNMT indicated poorer prognosis. Lower expression (blue line) of GLA indicated lower patient survival (*P* < 0.05). The correlation between PDEMRGs and GC prognosis showed that PDEMRGs contribute to the progression of GC (Figure 7B).

### External validation of the PDEMRGs using the online database

**Figure 8:**
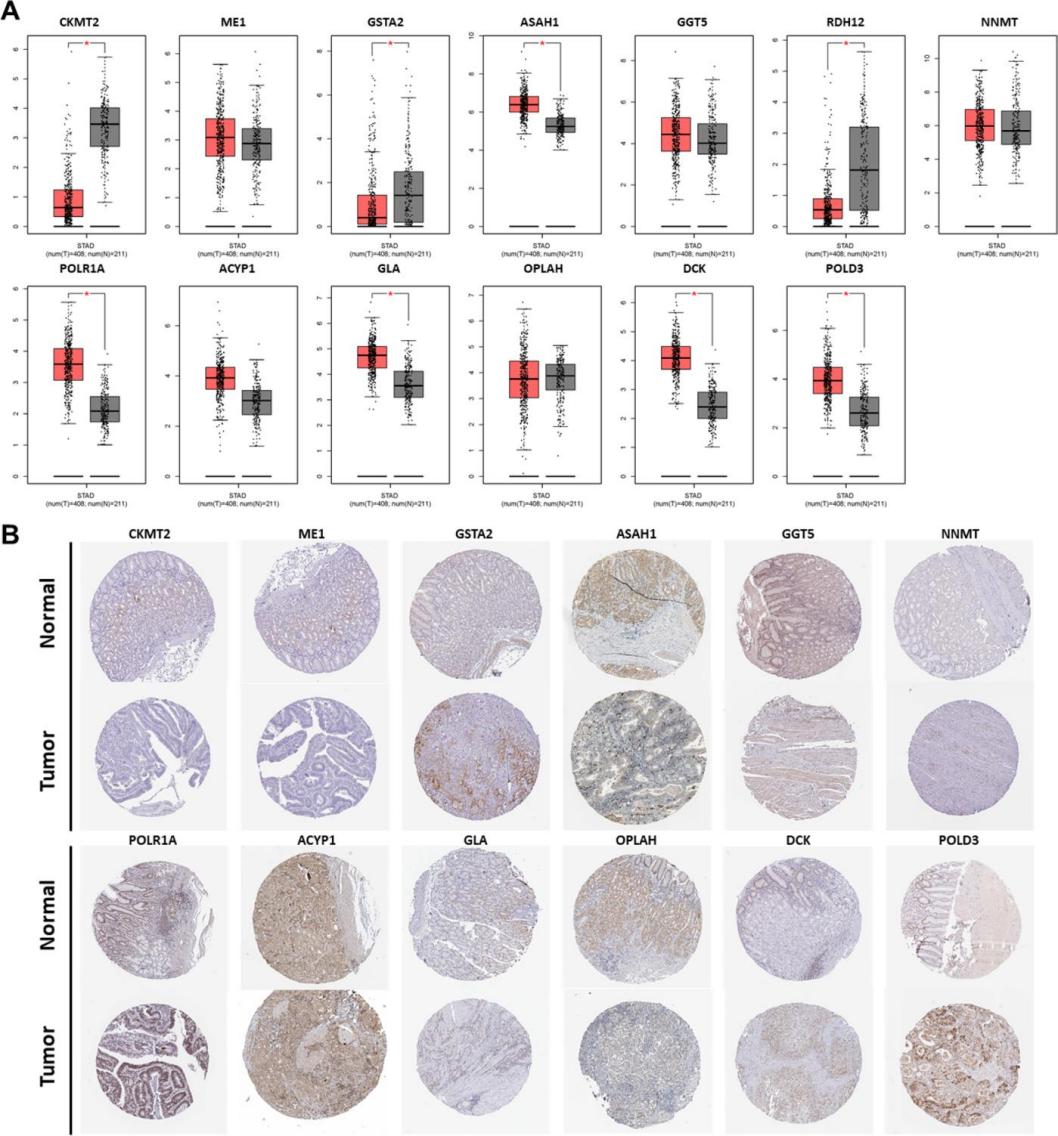
**Expression of the PDEMRGs.** (**A**) The mRNA expression levels of the PDEMRGs in GC and normal gastric tissue (*P < 0.05). Red represents GC and gray represents normal gastric tissue. (**B**) The representative protein expression of the PDEMRGs in GC and normal gastric tissue.

The Gene Expression Profiling Interactive Analysis database (GEPIA) corroborated the differences in gene expression between GC and normal gastric tissues. Boxplot showed most genes in the model had differences in GC mRNA expression compared with normal gastric tissues (*P* < 0.05) (Figure 8A). Genes such as CKMT2, GSTA2, and RDH12 were down-regulated, while POLR1A, ASAH1, GLA, DCK, and POLD3 were up-regulated. Representative protein expression was determined in the Human Protein Atlas (Figure 8B). The immunohistochemistry of GC genes was positive compared with normal gastric tissues, suggesting that the protein expression was increased. These results are consistent with the results of mRNA expression. However, RDH12 was not found in the database.

**Figure 9:**
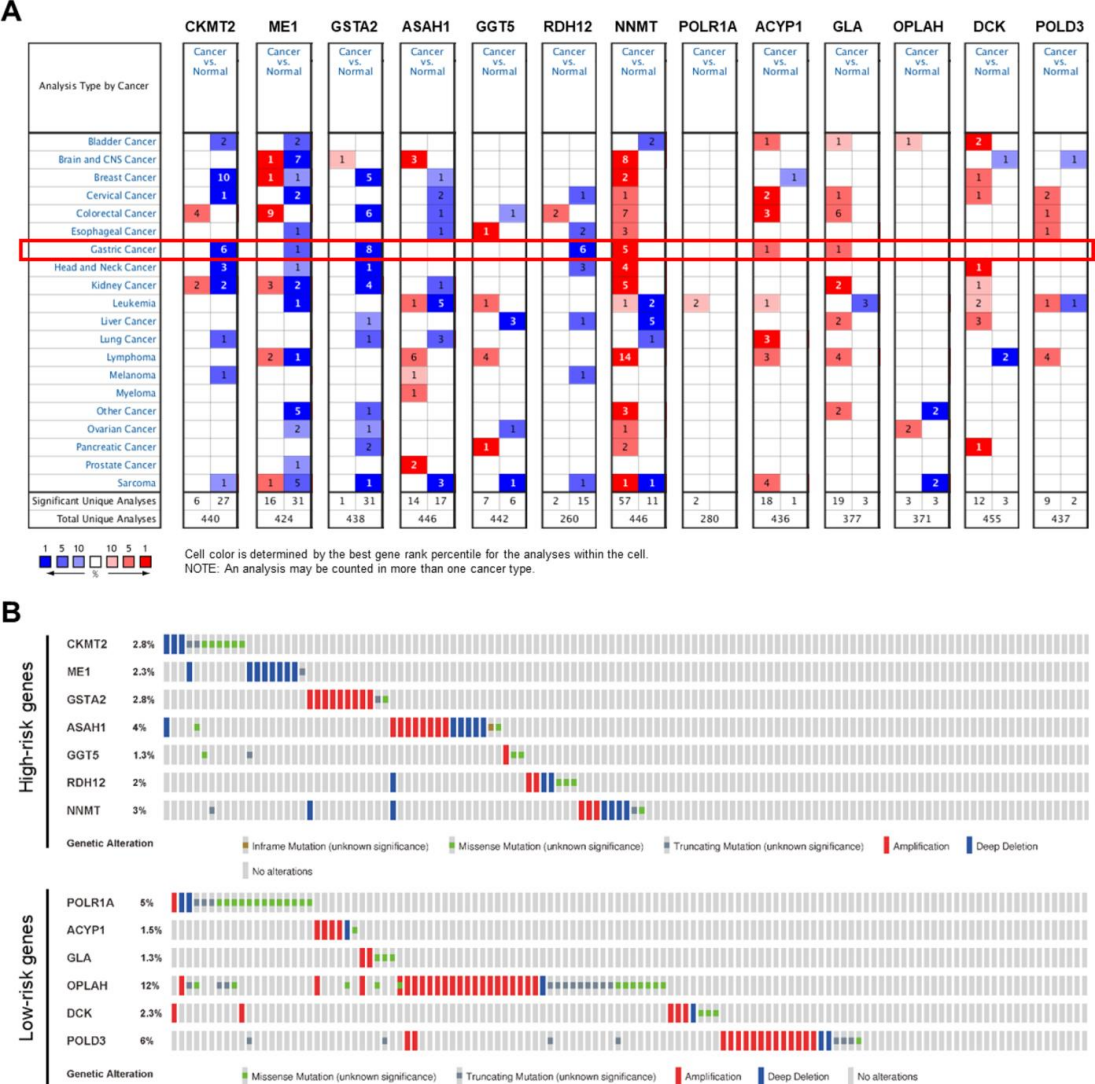
**Genetic alterations of the PDEMRGs.** (**A**) The expression profiles of the PDEMRGs in the Oncomine database. Red represents over-expressed; blue represents under-expressed. (**B**) Genetic alterations of the PDEMRGs from cBioportal for Cancer Genomics.

NNMT, ACYP1, and GLA were significantly over-expressed in GC, while CKMT2, ME1, GSTA2, and RDH12 were significantly under-expressed in the Oncomine database (Figure 9A). There is no mRNA expression of ASAH1, GGT5, POLR1A, OPLAH, DCK, and POLD3 in GC in the Oncomine database, but these genes have been confirmed to be over-expressed in GC both in the GEPIA and The Human Protein Atlas. Mutations in the form of amplification and deletion was expressed in the high-risk group, while amplification was expressed in the low-risk group. OPLAH had the most common genetic alterations (12%), and amplification was the most frequent genetic alteration (Figure 9B).

**Figure 10:**
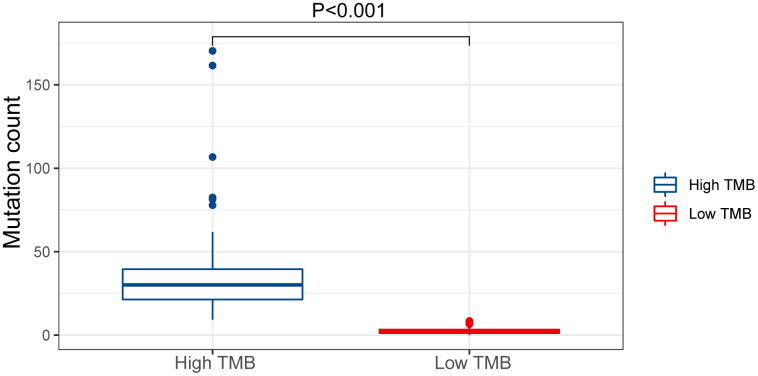
**Mutation count of GC patients in the low and high TMB groups of the TCGA cohort.**

We evaluated the tumor mutation burden (TMB) of the GC dataset and found the mutation count in the high TMB group was higher compared with the low-risk group (*p* < 0.05) (Figure 10). This result illustrates that our model can stratify patients for personalized treatment.

## DISCUSSION

The development of genome sequencing and combining prognosis-related genes with traditional parameters has advantages in predicting cancer [15, 16]. Metabolism-related research is a new research focus [7]. Recent studies have analyzed the association between metabolism-related genes, the risk of GC, and diagnostic value [17]. Wei Zheng found that single nucleotide polymorphisms (SNPs) in trace element-related metabolic genes were related to GC risk [18]. Therefore, the expression of PDEMRGs may predict the progression of GC and the prognosis of GC patients. We identified the PDEMRGs based on a training cohort and utilized the PDEMRGs to build a reliable model to predict the OS in GC patients, which we verified with a verification cohort.

Univariate and multivariate Cox regression analysis showed that this model was an independent prognostic factor independent of other clinical indices. The risk score derived with the model was more accurate than other clinical parameters in predicting OS. Nomogram analysis showed that this model combined with clinical indices (age, gender, grade, stage, and TNM) could be used to accurately predict the OS of GC patients at 1, 3, and 5 years. This may help plan short-term follow-up of individualized treatment, so that early intervention can improve prognosis.

GSEA analysis showed that abundant metabolic pathways were related to tumors, which confirmed the close relationship between the model and the metabolic system. Patients in the high-risk group were associated with arachidonic acid and cytochrome P450 metabolic pathways, while patients in the low-risk group were associated with pyrimidine, glyoxylic acid and dicarboxylates, aspartic acid and glutamate, alanine, cysteine, and methionine, fructose and mannose, and purine metabolic pathways. Interestingly, the low-risk group involved more diverse metabolic pathways, and they may benefit from metabolic-related treatments.

We also analyzed the clinical application of the model. The expression of several genes, including GGT5, NNMT and ME1, was increased in stage I and II GC, and there was a negative association with patient survival. This indicates that this model has high prognostic value, especially for the short-term survival of GC patients. The PDEMRGs in the prognostic risk model are associated with the occurrence and development of cancer. For instance, ME1, a well-known oncogene, promotes GC growth, lung metastasis, and peritoneal dissemination, and over- expression of ME1 correlates with shorter and disease-free GC survival [19]. In addition, cancer cells expressing NNMT can alter the epigenetic state and increase expression of pro-tumorigenic gene products [20].

DNA replication stress induced by oncogenes is considered a driving factor of tumorigenesis. Research has shown that POLD3 plays a unique role in the process of RS-induced DNA break repair, so targeting POLD3 could provide a priority opportunity to target cancer cells [21–23]. DCK negatively regulates the transcriptional activity of NRF2, resulting in a decrease in the expression of antioxidant genes, and negatively regulates intracellular redox homeostasis and ROS production. DCK has a negative regulatory effect on the proliferation and metastasis of pancreatic cancer cells. The low expression of DCK promoted NRF2-mediated antioxidant transcription, which enhanced drug resistance to gemcitabine [24]. OPLAH encodes the 5-oxoproline enzyme, which controls the synthesis and degradation of glutathione. Hypermethylation of OPLAH3 is a common feature of some tumors. Naumov et al. sequenced the genomes of 22 pairs of colorectal cancer (CRC) and adjacent tissues and found that OPLAH is the initiation gene of DNA methylation in CRC [25]. Roy et al. found that GLA promotes mitochondrial death and apoptosis and reduces hypoxia. It combines limitation of de novo fatty acid synthesis and the cholinergic anti-inflammatory pathway that confirms anticancer function [26]. Cao et al. reported that ACYP1 was lower in imatinib-resistant gastrointestinal stromal tumor T1 cells [27]. Silencing POLR1A can hinder G1-S cell cycle progression in p53-inactivated human cancer cell lines [28]. Guo et al. observed that in cervical squamous cell carcinoma (CSCC) tissues, RDH12 expression was reduced by 74.5%. The expression of RDH12 was negatively associated with tumor size and infiltration depth in cervical cancer [29]. Studies have confirmed that GGT5 gene amplification contributes to non-small cell lung cancer (NSCLC). Cells produce high levels of glutamate and promote glutamine metabolism [30]. Immunohistochemical staining was used to detect the expression of ASAH1 in 120 cases of non-special invasive ductal carcinoma (IDC-NOS). The expression of ASAH1 correlated with lymph node metastasis, suggesting that ASAH1 is a biomarker predictive of lymph node status [31]. Low expression of glutathione S-transferases (GSTs) in the liver reduces the detoxification of chemical carcinogens. GSTA2 was found to be in linkage disequilibrium in Caucasians [32]. It was concluded that CKMT2 was a key regulatory factor in the development of osteosarcoma, and significantly correlated with patient OS [33].

Mutations in the genome can alter gene expression [34]. Research by DeBerardinis and Chandel confirmed that glycolysis is correlated with activated oncogenes and mutated tumor suppressors [11]. When Laskowski and her colleagues studied aging complement factor H-deficient mice, they observed spontaneous hepatic tumor formation in more than 50% [35]. The results illustrate the interaction between aging, genetic mutation, and cancer. We believe that aging is closely related to gastric cancer. Additionally, Hamada et al. found that tumor mutation burden (TMB) is related to the emergence of new antigens [36]. Therefore, we checked whether this model reflected the TMB of GC patients. The results showed the high TMB group was significantly higher than the low TMB group. We found the overall probability of genetic alterations was higher in the low-risk group than in the high-risk group. These findings indicate that this model can be used in patients with different metabolic abnormalities, making individualized therapy strategies possible.

In summary, we used 13 PDEMRGs to build a risk model that accurately predicts the prognosis in patients with GC. In addition, this model reflects the dysregulated metabolic microenvironment in tumor patients, and provides biomarkers for metabolic treatment of these patients. However, further in vitro and in vivo experiments are needed to validate the results of this research.

## MATERIALS AND METHODS

### Data collection

Transcriptome data and the relevant clinical data were downloaded from TCGA (https://portal.gdc.cancer.gov/) and GEO (https://www.ncbi.nlm.nih.gov/geo/). The somatic mutation data were obtained from TCGA. The candidate metabolic gene sets were searched from “c2.cp.kegg. v7.0. symbols” of Gene Set Enrichment Analysis (GSEA).

### Identification of differentially expressed metabolism-related genes

Wilcoxon signed-rank test was used to analyze the differences of 745 annotated MRGs with protein-coding functions. Screening condition: false-discovery rate [FDR] < 0.05, log2 fold-change [FC] > 0.5.

### Establishment of experimental model

We used univariate Cox analysis to initially identify potential PDEMRGs. Lasso penalty Cox regression analysis [37] was used for confirmation. The penalized maximum likelihood estimator with 1000-fold cross validation was used to construct the prognostic risk model. The expression values of PDEMRGs were weighted by the regression coefficient of the Cox regression model to calculate the risk score of every patient. Risk score = (Coefficient_mRNA1_×mRNA1 expression) + (Coefficient_mRNA2_×mRNA2 expression) + ⋯ + (Coefficient_mRNAn_× mRNAn expression). Taking the median risk score of the training cohort as the cut-off value, all patients with GC were divided into a high-risk group and a low-risk group. R packages “survival” [38] and “survminer” were performed to compare the survival differences between the high-and low-risk group, and a significant *p*-value was obtained. The verification cohort was used for verification.

### Independence of the PDEMRGs

Univariate and multivariate Cox regression analysis were performed to analyze the independent prognosis of GC patients with forwarding stepwise procedure. *P* < 0.05 indicated statistical significance. The “SurvivalROC” [39] of R package was used to determine the prognostic value of the risk score. The nomogram [40] was constructed by including all independent prognostic factors to predict the survival of GC patients at 1, 3, and 5 years.

### Gene set enrichment analyses

GSEA v4.0.1 software (https://www.gsea-msigdb.org/gsea/login.jsp) was run to reveal potential biological pathways and mechanisms in the KEGG. *P* < 0.05 was considered statistically significant.

### External verification of PDEMRGs

To verify the expression of PDEMRGs in this model, the mRNA level was validated by Gene Expression Profiling Interactive Analysis database (GEPIA, http://gepia.cancer-pku.cn/) and the Oncomine database (https://www.oncomine.org/resource/main.html). The Human Protein Atlas database (http://www.proteinatlas.org) was used for the protein level. GEPIA was also used to determine the pathological stage and survival of PMRGs in this model. The genetic alterations of PMRGs in this model were evaluated by cBioportal for Cancer Genomics (http://www.cbioportal.org/).

### Statistical analysis

All statistical analyses were run through R software version 3.6.1 (https://www.r-project.org/). Differences between variables were evaluated using independent t-tests. Log-rank test was used to compare the high-risk with the low-risk group in the Kaplan-Meier curve. Qualitative variables were compared by Pearson χ^2^ test or Fisher's exact test. A two-sided *P*<0.05 was considered statistically significant.
